# Acoustic Impedance Evaluation of the Polymer–Polymer Hybrid Composites as Insulator Building Materials

**DOI:** 10.3390/polym15163479

**Published:** 2023-08-20

**Authors:** Besma M. Fahad, Rand Salih Farhan Al-jadiri

**Affiliations:** Materials Engineering Department, College of Engineering, Mustansiriyah University, Baghdad 10052, Iraq

**Keywords:** composite materials, polymeric composite, hybrid composites, ultrasonic test, acoustic impedance

## Abstract

Acoustic energy dissipates in multi-phase or multi-boundary materials. Hybrid composites are described as multi-phase with many interfaces between their materials. The current research proposes the study of the acoustic behavior of polymeric hybrid composites by estimating the time, velocity, and hybrid composite acoustic impedance. Two groups of hybrid composites were prepared, including unsaturated polyester with PMMA, except one with HDPE and the other with PS. Each group had 28%, 35%, and 40% weight fractions. An ultrasonic test measured the time to determine the velocity and then the acoustic impedance later. The results showed that increasing the weight fraction will increase the density with respect to the density of the reinforcing material. Different ultrasonic times were obtained with increasing weight fractions. As the weight fraction of PS increased, the time increased; unlike the velocity, it decreased but increased with density. In contrast, this behavior was changed if the hybrid had PE. The highest acoustic impedance was at 28% UP/PMMA + PS. In conclusion, UP/PMMA + PS can dissipate ultrasonic waves more than UP/PMMA + PE.

## 1. Introduction

There is no universally accepted definition of composite materials. Definitions in the literature differ widely. The word “composite” is used in the dictionary and everyday conversation to describe anything consisting of several parts or elements [1]. Thus, a material with two or more distinct constituent materials or phases may be considered composite [2,3]. Polymeric composites combine a resin with a reinforcing agent to increase the plastic matrix’s characteristics for various applications [4,5]. Reinforcing material describes a phase used as a filler or reinforcement incorporated into a polymer matrix. Polymers have gained particular attention due to their simplicity of processing, lightweight nature, low cost, and a vast range of options and qualities [2,6]. The addition of fillers to a polymer matrix may enhance its overall characteristics and, in certain situations, offer additional functionalities like sound insulation [7].

Hybrid composites adapt these perspectives in which various material combinations are employed to optimize efficiency. Hybrids may, in theory, be made out of any mixture of various materials [8]. Hybridization tries to create a new material with the benefits of its constituents but without drawbacks [9]. Polymers have emerged as a critical component in composites in recent years. Many scientists are searching for new and alternative metals because of the paucity of metals. Various factors, like composites’ low cost, simplicity of fabrication, and good mechanical characteristics, make them desirable materials. Unsaturated polyester stands out among these options because of its dimensional stability, inexpensive cost, and lightweight nature [10]. It belongs to the category of thermosetting polymers. They provide attractive benefits in terms of their extensive applications [11,12]. Bergant et al. employed the pulse-echo ultrasonic C-scan technique to look for imperfections in a composite plate during manufacturing. Epoxy matrix infusion was used to create a glass-fiber-reinforced epoxy composite (VI).

Several artificial flaws of various shapes, volumes, and depths were inserted into test plates. Air was introduced via a tiny, unsealed spot in the vacuum membrane throughout the specimen processing with VI. PVC, aluminum chips, and aluminum foils were put between layers at the appropriate positions. The 4D C-scan approach was utilized to examine the defects in the plate using the ultrasonic immersion system with water coupling. Various frequencies and gate settings were employed to identify the ideal variables for detecting defects. The C-scan images accurately located and detected aluminum chips and PVC foils. Additionally, individual layers’ porosity may be detected. The thinness of the metal foils, 0.04 mm, was undetectable by this approach [13].

Industrial wastes from various industries were collected and employed as particle reinforcement in an unsaturated polyester matrix and polypropylene by Nimmagadda et al. The results of the composite were examined for their dielectric characteristics. The findings showed that composites treated with coupling agents have increased dielectric strength due to the better compatibility between the matrix and reinforcement interface. Additionally, the findings showed that polypropylene reinforced with industrial waste has greater dielectric strength than polyester reinforcement [14]. Taheri and Hassen employed phased array ultrasonic testing (PAUT) to find flaws in composite materials. Wind-turbine-blade glass-fiber-reinforced plastic (GFRP) plates were employed in the research. The thicknesses of the GFRP samples ranged from 4 to 25 mm. Different-sized holes, up to 25 mm in diameter, were drilled into one side of the sample to investigate the sensitivity of flaw detection using PAUT and SEUT. The findings demonstrated that PAUT can identify flaws with a size of 0.8 mm and a penetration depth of up to 25 mm. The resulting signals outperformed the conventional ultrasonic method in terms of their properties [15].

Khoramishad and Mousavi used the finite element method to predict the performance of hybrid composites under impact loading at a macro scale. Glass and carbon fibers were used to create the laminates of the hybrid composite. The ballistic limitation was calculated using the Lambert–Jonas empirical model and the least-squares approach. The outcomes of the numerical simulations were verified against those of previously reported experiments. The average percentage variance between the simulated and experimental findings was just 3.22%, indicating good agreement [16].

The efficiency of ultrasonic damage detection for low-velocity impacts on various composite laminates at varying energy levels was studied by Papa et al. Despite the parameter change, the latter method guaranteed that it could provide data on the delamination’s shape and size. It was even effective for testing the effect of various elements on the dynamic behavior of the examined composites. Ultrasound waves were diffused and detected using the pulse-echo technique with facing-array transducers (f 1/4 5 MHz). The findings aided in elucidating the processes, initiation, and spread of damage [17]. Others studied the sound insulation using different devices (not ultrasonic), such as Borlea et al., who measured the sound absorption coefficient on a frequency basis for particular composites consisting of recycled rubber, pine sawdust, and a polyurethane binder [18]. Bratu et al., investigated the proportion of grain steelwork slag, glass fiber wastes, wood waste, and oil seed ash waste as reinforcements with formaldehyde resin matrix to measure the sound absorbing capacity [19]. Gliscinska et al., studied thermoplastic sound-absorbing composites manufactured from mix flax fibers with polylactide fibers [20]. In previous studies, authors have studied different properties of composites or hybrids. They used ultrasonic techniques to detect defects or estimate the dynamic properties of the composites. However, this work aimed to utilize ultrasonic technology to measure the acoustic impedance of hybrid composites made from different polymers. This study aims to spot the importance of introducing a multi-interface in a composite with respect to the nature of the reinforcing material that will ensure the good dissipation of sound and, as a result, a good insulation material. The novelty of this work is twofold: the first is the application of a hybrid composite, which is considered as a multi-interface composite and as insulating building material; and the second is the measurement of the acoustic impedance by the ultrasonic technique, which has been studied little in other research (they all measure the sound absorption in the material only). The purpose of selecting different polymers to prepare hybrid composites is twofold. The first is to keep it lightweight at a low cost. The second is to manufacture hybrid composites from different polymers to utilize the fundamental fact that polymers differ according to their structures and manufacturing techniques. This will create a long path for the acoustic wave to dissipate energy by making multimedia that act as acoustic impedances, giving better insulation. The unsaturated polyester (UP) was selected to be reinforced with polymethyl methacrylate (PMMA) and hybridized with either high-density polyethylene particles (PE) or polystyrene (PS). In addition, the acoustic impedance was also studied for the hybrid composite when tested by the ultrasonic technique.

## 2. Materials and Methods

Polymers can be used in many applications, such as acoustic, thermal, and electrical insulators. The polymer used in this work was unsaturated polyester (UP) (as a matrix) Table 1 expresses the important technical data of the unsaturated polyester. Intermid Petrochemical Industrial provided the polymers that were bought from the local market, (IPI).

The polymer was composed of an unsaturated polyester resin, an accelerator (cobalt naphthalate), and a hardener (Methyl Ethyl Ketone Peroxide (MEKP)). The hardener was applied to the resin. On the other hand, the accelerator was mixed into the resin before the hardener was added to speed up the reaction between the two materials. In this procedure, the liquid resin was transformed into a solid.

**Figure 1:**
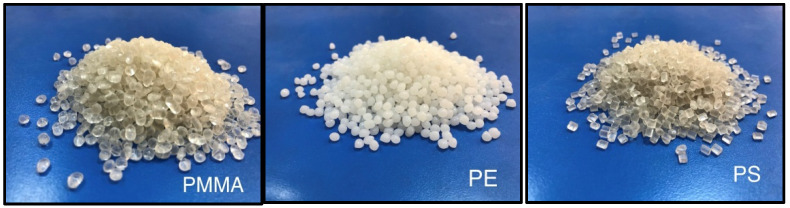
Solid polymeric reinforcements.

The matrix was in a liquid state, while the reinforcements were solid polymeric materials with an almost spherical shape (2 mm diameter), made of polystyrene (PS), poly methyl methacrylate (PMMA), and high-density polyethylene (HDPE or PE), as shown in Figure 1.

### 2.1. Specimen Preparation

The formation of hybrid composites began with the preparation of the required materials. It consisted of resin, reinforcement, a mold, and a mold release agent. Selected weight fractions were calculated by weighing the ingredients on a precise balance. There were two sets of hybrid samples made. Unsaturated polyester reinforced with PMMA and polyethylene (PE) composed the first group, whereas unsaturated polyester reinforced with PMMA and polystyrene (PS) composed the second. Three weight fractions of 28%, 35%, and 40% were applied to generate each sample.

The specimen was de-molded using a cubic metal mold that measures 5 × 5 × 5 cm and includes a detachable base for easy removal. The mold was cleaned, and a releasing agent was utilized on the inside surface. The samples were produced using a hand lay-up technique. The matrix material selected for this study was unsaturated polyester. The accelerator, Cobalt Naphthalate, was blended with the substance, resulting in a transparent solution that subsequently acquired a pink hue. The hardener, MEKP, was later incorporated into the mixture.

The exothermic reaction was regulated, and the formation of internal stresses or bubbles was prevented by adding each accelerator and hardener at precise percentages. The polymers were combined until they became homogenous. A fixed quantity of PMMA was supplemented with PS/PE reinforcement weighing 2, 4, and 6 g to achieve weight fractions of 28%, 35%, and 40%, respectively.

**Figure 2:**
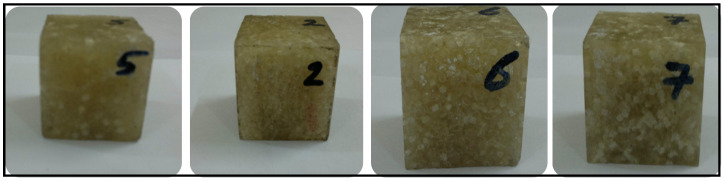
Hybrid composite specimens.

The composite specimens were subjected to mixing in order to attain an optimal distribution of the mixture. The hybrid composites were cured at ambient conditions for 24 h until complete hardening was achieved. Following the curing process, the specimens were removed from their molds, and their weights were measured. The hybrid specimens are depicted in Figure 2. The density of cube specimens was calculated by dividing its weight by its volume.

### 2.2. Ultrasonic Technique

**Figure 3:**
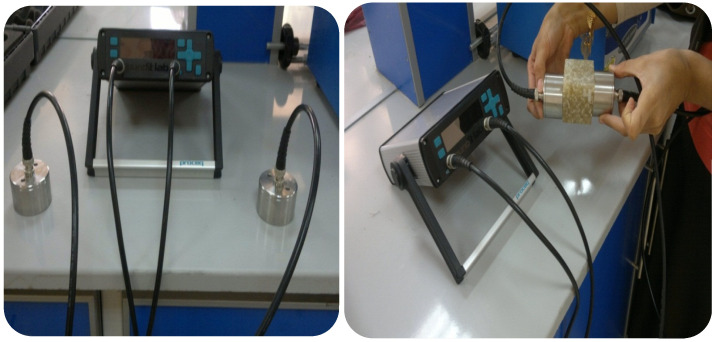
Ultrasonic test device.

Ultrasonic testing is a prevalent non-destructive testing method employed to evaluate materials. Ultrasonic technology finds its application in assessing the quality of partially processed material through inspection. This technique is utilized to identify internal flaws in materials, and it has the added capability of detecting minor surface cracks and assessing sonic parameters [21,22,23]. The examination can be conducted utilizing either a singular transducer in a pulse-echo configuration or two transducers in a through-transmission design. Coupling the transducer(s) to the structure through a solid or liquid medium is necessary to address the significant impedance mismatch between solid materials and air. The present study conducted an ultrasonic test utilizing a pair of transducers in through-transmission mode, as depicted in Figure 3. For accurate readings, the surface of the specimen must be both clean and smooth when manual testing is conducted. Subsequently, a thin gel coating could be affixed between the transducer and the structure. The experiment was conducted at ambient temperature for all hybrid composite specimens. All opposing sides of the cubic specimens were evaluated using ultrasonics to verify the homogeneity of the specimens. Instead of measuring velocity directly, the ultrasonic method detects a transit time delay. The length is involved in the velocity and has to be measured independently. Volume or weight fractions and the kind of reinforcement affect the velocity of ultrasonic waves in composites. The acoustic impedance depends on the velocity of the ultrasonic wave and density of the medium through which it is transmitted. The velocity, density, and acoustic impedance were calculated for all hybrid composites. As is well known, the velocity (V) is the relation between the length of the specimen (l) and ultrasonic time (t) according to Equation (1):

V = l/t(1)

The density (ρ) is the mass (w) per unit volume (v) of the specimen, as in the relation:

ρ = w/v(2)

The acoustic impedance (Z) can be measured from the following equation:

Z = ρ·V(3)

## 3. Results and Discussion

**Figure 4:**
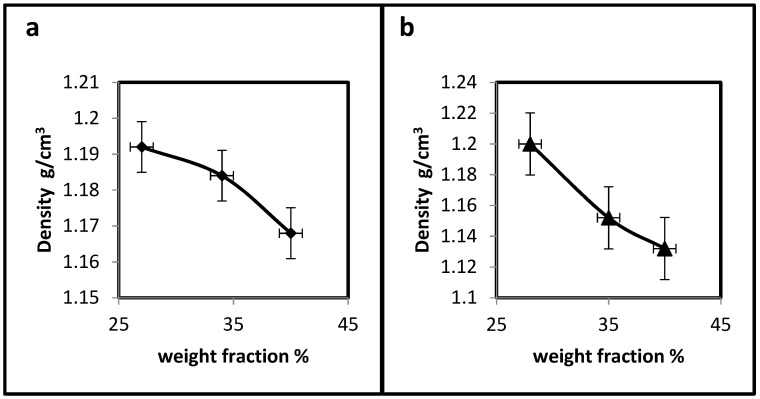
Effect of weight fraction on the hybrid composites density. (**a**)-UP/PMMA + PS; (**b**)-UP/PMMA + PE.

Sound waves are elastic waves that can be transmitted through solid media. Ultrasonic waves are mechanical vibrations that vibrate the actual particles of matter, so different materials have different wavelengths [24,25]. Since ultrasound is related to the media transfer, the material’s density will affect the wave energy. As the percent of PS or PE increases in the hybrid composites, the density decreases, as illustrated in Figure 4a,b. This decrease in density is a logical result since the composites or hybrid composites acquire their properties from their components. The density of PMMA is (1.18 g/cm^3^) higher than both PS and PE densities (1.07 and 0.88–0.96 g/cm^3^, respectively), so the addition of either PS or PE to the UP/PMMA composite will decrease its density as the weight fraction increases.

**Figure 5:**
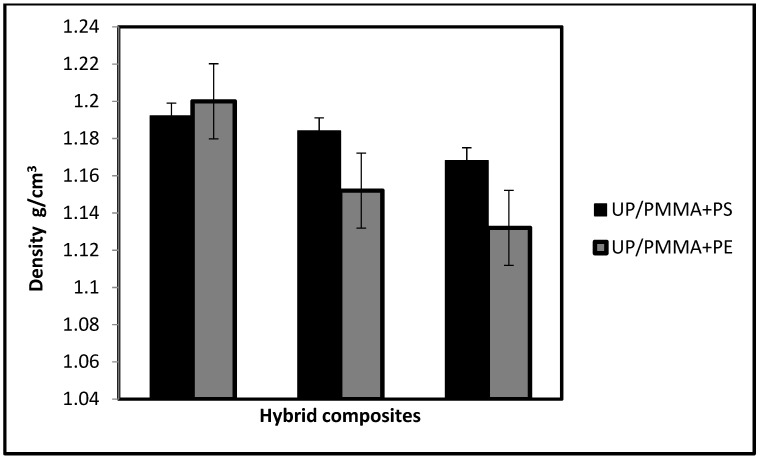
Comparison between the densities of different hybrid composites.

When comparing both hybrid groups, the density of UP/PMMA + PS is higher than UP/PMMA + PE except for the 28% weight fraction, as shown in Figure 5. Composite materials can behave differently in testing using ultrasonic waves according to the reinforcing materials, percent, geometry of reinforcing, boundary, and interfaces between the matrix and reinforcing materials. Other factors could influence the propagation of sound waves [26].

The effective dissipation of wave energy serves as a measure of an insulator’s efficiency. Besides the hybridization, small solid spheres were chosen to reinforce unsaturated polyester to expand the path of acoustic waves, which will scatter their energy and improve the polyester’s insulation properties. Table 2 clarifies the variation in ultrasonic time with increasing weight fractions, which indicates that increasing the quantity of hybridization besides the type of materials will influence the time of the passing wave. The addition of PS had a greater effect on the behavior of the ultrasonic wave, i.e., the time increased as the weight fraction increased, unlike the addition of PE, which showed almost constant behavior when the weight fraction increased. This agrees with [27] that composite materials can behave differently according to the nature of the reinforcing.

**Figure 6:**
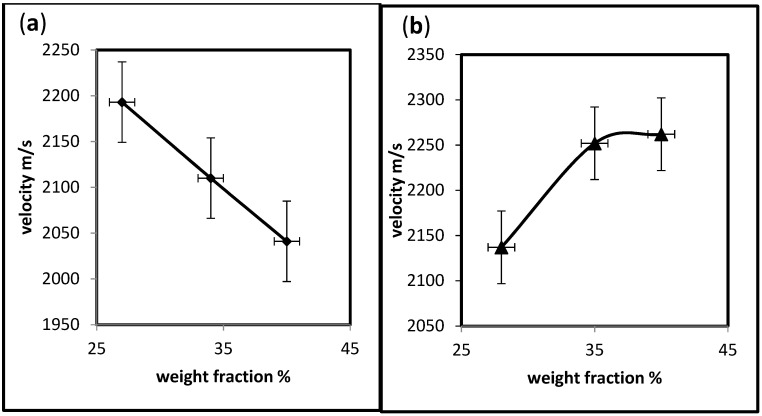
Effect of weight fraction on the velocity through hybrid composites. (**a**)-UP/PMMA + PS; (**b**)-UP/PMMA + PE.

**Figure 7:**
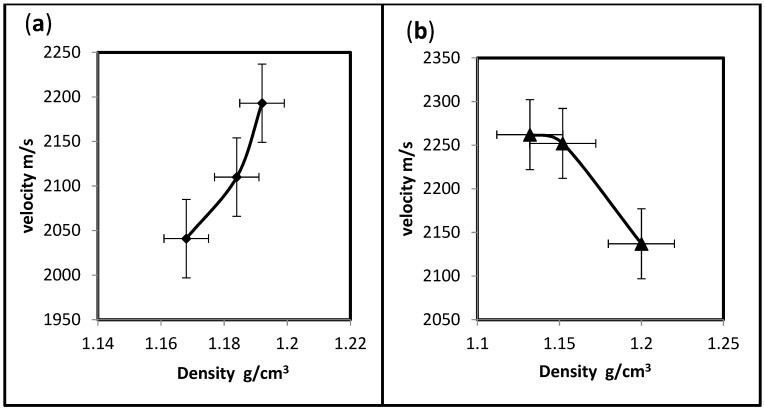
Effect of density on the velocity through the hybrid composites. (**a**)-UP/PMMA + PS; (**b**)-UP/PMMA + PE.

Composites are anisotropic materials, making them very difficult to inspect, so again, the velocity acts according to the type of reinforcing materials. Some polymers are semi-crystalline polymers, while others are glassy polymers. It decreases as the weight fraction increases when the hybrid contains PS and increases when PE is included. Unlike the behavior of velocity with density, it increases if the hybrid has PS and decreases if it has PE. This result corresponds with the fact that the denser the material, the faster it transmits the ultrasound wave. This confirms that the material’s nature and structure influence the ultrasound wave’s velocity, not only its percent, as demonstrated in Figure 6 and Figure 7a,b.

**Figure 8:**
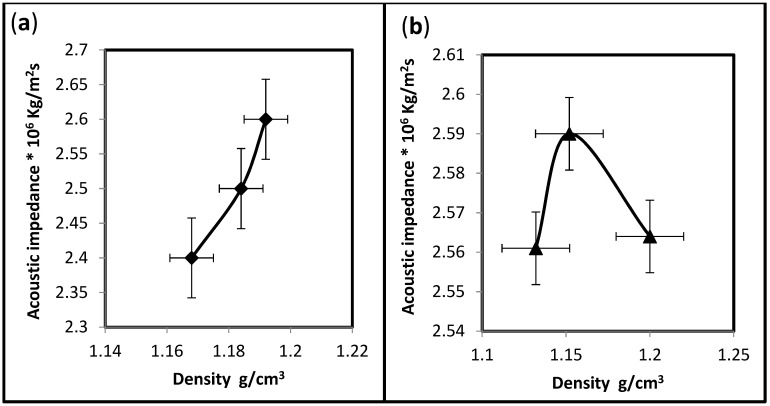
Effect of density on the acoustic impedance of hybrid composites. (**a**)-UP/PMMA + PS; (**b**)-UP/PMMA + PE.

The acoustic impedance is a function of material density and the velocity transmitted. In composites, the nature and percent of the reinforcing materials can determine the density, and different reinforcing materials can create many grain boundaries or interfaces. When ultrasound meets a boundary between two media, some energy is reflected and some travels on, i.e., is transmitted [28]. It is crucial to ensure that the ultrasonic probe is fixed well on the surface of the specimen by a good coupling agent; in addition, the specimen should have no cracks that could result in incorrect readings. Figure 8a,b demonstrate the different behavior of the acoustic impedance of both hybrid composites, indicating that the polymers can behave differently against ultrasound waves. The acoustic impedance of UP/PMMA-PS hybrids increased as the density increased. In contrast, UP/PMMA-PE hybrids seem to have a preferable density, which means that less or more than this density will reduce the acoustic impedance. However, its relative values may indicate constant behavior.

**Figure 9:**
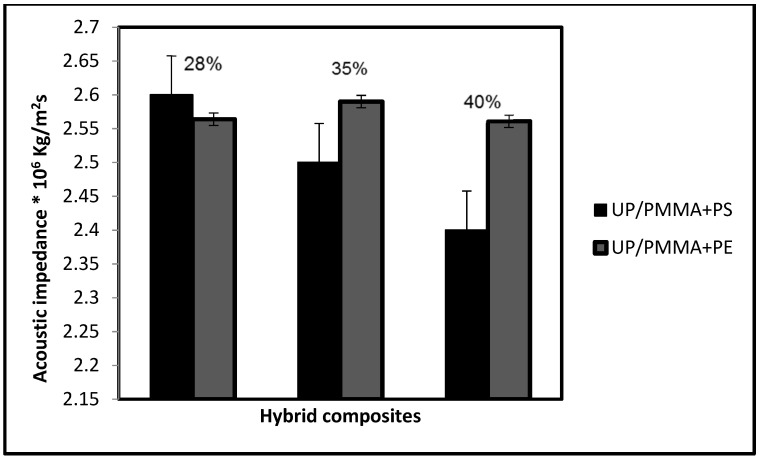
Comparison between the acoustic impedance of both hybrid composites with weight fraction.

When comparing the acoustic impedance behavior of both hybrids with respect to the weight fraction, it appears that 28% UP/PMMA + PS has the highest acoustic impedance, as shown in Figure 9.

To understand the best hybrid to isolate sound, it is necessary to study how an ultrasound wave travels through the specimen by finding the effect of acoustic impedance on time. Table 3 indicates the two cases of hybrids. UP/PMMA + PS seems to be a better insulator than UP/PMMA + PE because, as the acoustic impedance varies in value, the time necessary to travel through the specimen will be altered. In other words, when the percentage of PS increases, it becomes more dominant than the other components. Subsequently, this increase will affect the behavior, i.e., the acoustic impedance will decrease, and the wave time will increase, which means that the wave will be scattered according to the quantity of the hybrid material and the amount of the interfaces generated within the composite. Unlike the addition of PE, which gives almost constant behavior whatever its percent, it does not create the necessary interfaces that could impede the wave path and improve the insulation properties. The percentage of energy transmitted and reflected depends on the acoustic impedance of the two media (the matrix and reinforcing). In the case of hybrid composites, the wave in the first reinforcing material (PMMA) will split at the interface between the matrix and the first reinforcement into transmitted and reflected waves. The transmitted component is again divided at the interface between the matrix and the second reinforcement (PS or PE). The result is a sequence of reflected waves of both directions between the interface in both reflected and transmitted components that cause the dissipation of the ultrasound energy.

## 4. Conclusions

Undeniably, the quantity of hybridization, besides the type of materials, will directly influence the properties of an insulator. The density of UP/PMMA + PS is higher than that of UP/PMMA + PE, except for the 28% weight fraction. There is a variation in ultrasonic time with increasing weight fractions. As the PS weight fraction increases, the time increases, unlike the addition of PE, which shows almost constant behavior when the weight fraction increases. Ultrasonic velocity decreases as the weight fraction increases when the hybrid contains PS and increases when PE is included. Unlike the behavior of velocity with density, it increases if the hybrid has PS and decreases if it has PE. The 28% UP/PMMA + PS has the highest acoustic impedance, indicating that UP/PMMA + PS is a better insulator than UP/PMMA + PE. This brings to light the fact that composite materials composed of two or more different kinds of components give rise to a new material with a combination of the properties of both kinds of components that give the opportunity to utilize the materials in classrooms, conference halls, hospitals …, etc., in other words, as building insulating materials.

## Figures and Tables

**Table 1:** Technical data of unsaturated polyester.

Property	Value
Specific gravity @25 °C	1.10–1.18
Viscosity @25 °C	300–450 mPa.s
Appearance	pink
Molding information	Hand lay-up and spray up
Main applications	General purpose

**Table 2:** Relation of time and weight fractions of hybrid composites.

UP/PMMA + PS		UP/PMMA + PE	
wt%	Time	wt%	Time
28	22.8	28	23.4
35	23.7	35	22.2
40	24.5	40	22.1

**Table 3:** The relation between time and the acoustic impedance of hybrid composites.

UP/PMMA + PS		UP/PMMA + PE	
Time	Z ∗ 10 ^6^	Time	Z ∗ 10 ^6^
22.8	2.6	23.4	2.564
23.7	2.5	22.2	2.590
24.5	2.4	22.1	2.561

## Data Availability

Not applicable.
